# The role of microglia in neuronal and cognitive function during high altitude acclimatization

**DOI:** 10.1038/s41598-024-69694-9

**Published:** 2024-08-16

**Authors:** Kathleen Hatch, Fritz Lischka, Mengfan Wang, Xiufen Xu, Cheryl D. Stimpson, Tara Barvir, Nathan P. Cramer, Daniel P. Perl, Guoqiang Yu, Caroline A. Browne, Dara L. Dickstein, Zygmunt Galdzicki

**Affiliations:** 1 Neuroscience Graduate Program, Uniformed Services University of the Health Sciences, Bethesda, MD 20814 USA; 2 The Henry M. Jackson Foundation for the Advancement of Military Medicine Inc. (HJF), 6720A Rockledge Drive, Bethesda, MD 20817 USA; 3 Department of Anatomy, Physiology and Genetics, Uniformed Services University of the Health Sciences, Bethesda, MD 20814 USA; 4 Department of Pathology, Uniformed Services University of the Health Sciences, Bethesda, MD 20814 USA; 5 Department of Pharmacology and Molecular Therapeutics, Uniformed Services University of the Health Sciences, Bethesda, MD 20814 USA; 6 Department of Electrical and Computer Engineering, Virginia Tech, Arlington, VA USA; 7 Present Address: Department of Anatomy and Neurobiology, University of Maryland School of Medicine, 655 W. Baltimore Street, Baltimore, MD 21201 USA

**Keywords:** High altitude, Microglia, Neurovasculature, Microglial heterogeneity, Microglia, Long-term potentiation, Fear conditioning, Neuro-vascular interactions

## Abstract

Due to their interactions with the neurovasculature, microglia are implicated in maladaptive responses to hypobaric hypoxia at high altitude (HA). To explore these interactions at HA, pharmacological depletion of microglia with the colony-stimulating factor-1 receptor inhibitor, PLX5622, was employed in male C57BL/6J mice maintained at HA or sea level (SL) for 3-weeks, followed by assessment of ex-vivo hippocampal long-term potentiation (LTP), fear memory recall and microglial dynamics/physiology. Our findings revealed that microglia depletion decreased LTP and reduced glucose levels by 25% at SL but did not affect fear memory recall. At HA, the absence of microglia did not significantly alter HA associated deficits in fear memory or HA mediated decreases in peripheral glucose levels. In regard to microglial dynamics in the cortex, HA enhanced microglial surveillance activity, ablation of microglia resulted in increased chemotactic responses and decreased microglia tip proliferation during ball formation. In contrast, vessel ablation increased cortical microglia tip path tortuosity. In the hippocampus, changes in microglial dynamics were only observed in response to vessel ablation following HA. As the hippocampus is critical for learning and memory, poor hippocampal microglial context-dependent adaptation may be responsible for some of the enduring neurological deficits associated with HA.

## Introduction

High altitude (HA) is characterized by hypobaric hypoxia, reducing oxygen availability for blood and tissue absorption with serious implications for cardiovascular, metabolic and cognitive functioning^1–5^. Mechanisms of chronic HA adaptation involve many molecular pathways including oxidative stress, inflammation, protein kinase activation, and hypoxia signaling^6^. Previous studies in murine models of chronic HA (~ 12-weeks) highlight alterations in myelination, inflammation, vascularization, synaptic protein expression^6–9^_,_ hippocampal dependent memory, and transcriptional changes in inflammatory, angiogenic and metabolic pathways^6^.

These alterations may be mediated in part by changes in context-dependent activity of microglia and their interactions with neurons and the surrounding neurovasculature. While initial activation of microglia is thought to be neuroprotective and preserve synaptic plasticity^10^, prolonged activation has been shown to be detrimental. Under inflammatory conditions, microglia shift to aerobic glycolysis and stabilize hypoxia-inducible factor-1α (HIF-1α), producing proinflammatory cytokines, such as TNF-α and IL-1β^11,12^, that contribute to inhibition of long-term potentiation (LTP), thereby diminishing synaptic transmission within a neuronal network^13–15^. Crosstalk between microglia and neurons can also affect microglial morphology, with the induction of LTP causing an increase in the number of microglial processes and increased contact duration of microglial processes with dendritic spines^16^. Neuronal hyperexcitability can lead to excitotoxicity and disruption of local ATP microgradients which in turn negatively impact microglial motility and phagocytic efficiency, triggering an uncoupling of critical phagocytosis apoptosis mechanisms^17,18^. Importantly, while microglia promote synaptic activity and enhance neuronal synchrony during healthy homeostatic surveillance activity, inflammatory activation of microglia actually contributes to impaired network synchronization, suggesting a role of inflammation and immune response in cognitive functioning^19^. Under healthy conditions, not only do microglia phagocytose dying neurons, prune synapses and produce cytokines/chemokines to promote neuronal health and communication, microglia also modulate neuronal activity primarily by suppressing neuronal hyperactivation^20,21^. Thus, microglia contribute to maintenance of homeostatic state through the neurophysiological and structural changes in the synaptic activity required to facilitate learning and memory^22^. The negative feedback control of microglia over synaptic activity is region-specific and relies on microglia sensing extracellular ATP and converting it to adenosine, which then acts at neuronal synapses to increase inhibitory tone^20^. Microglia also rescue neurons from excitotoxicity by preventing excess depolarization through migration of microglial processes towards swollen axons where microglia-axon contact facilitates removal of debris and membrane repolarization back to resting potential, thus preventing neuronal damage due to hyperactivity^13,17,23^. Activated microglia following injury along a graded continuum of insult use similar molecular mechanisms as those employed during development, to target and displace inhibitory synapses in cortical neurons to promote neuroprotection and mediate presynaptic stripping^14,24,25^. HA impacts the vascular environment of the brain triggering angiogenesis, vascular edema, and compromising blood–brain barrier^6^. Interactions of microglia with vasculature have been shown to be critical for microglia colonization and its territorial function in the brain, vascular performance, and blood brain barrier permeability, and unique populations of “juxtavascular” microglia (situated close to blood vessels) have been shown to exhibit unique functional patterns^26–28^. However, the impact of HA on neurovasculature and the context-dependent role of microglia in these processes have not been addressed yet.

**Figure 1:**
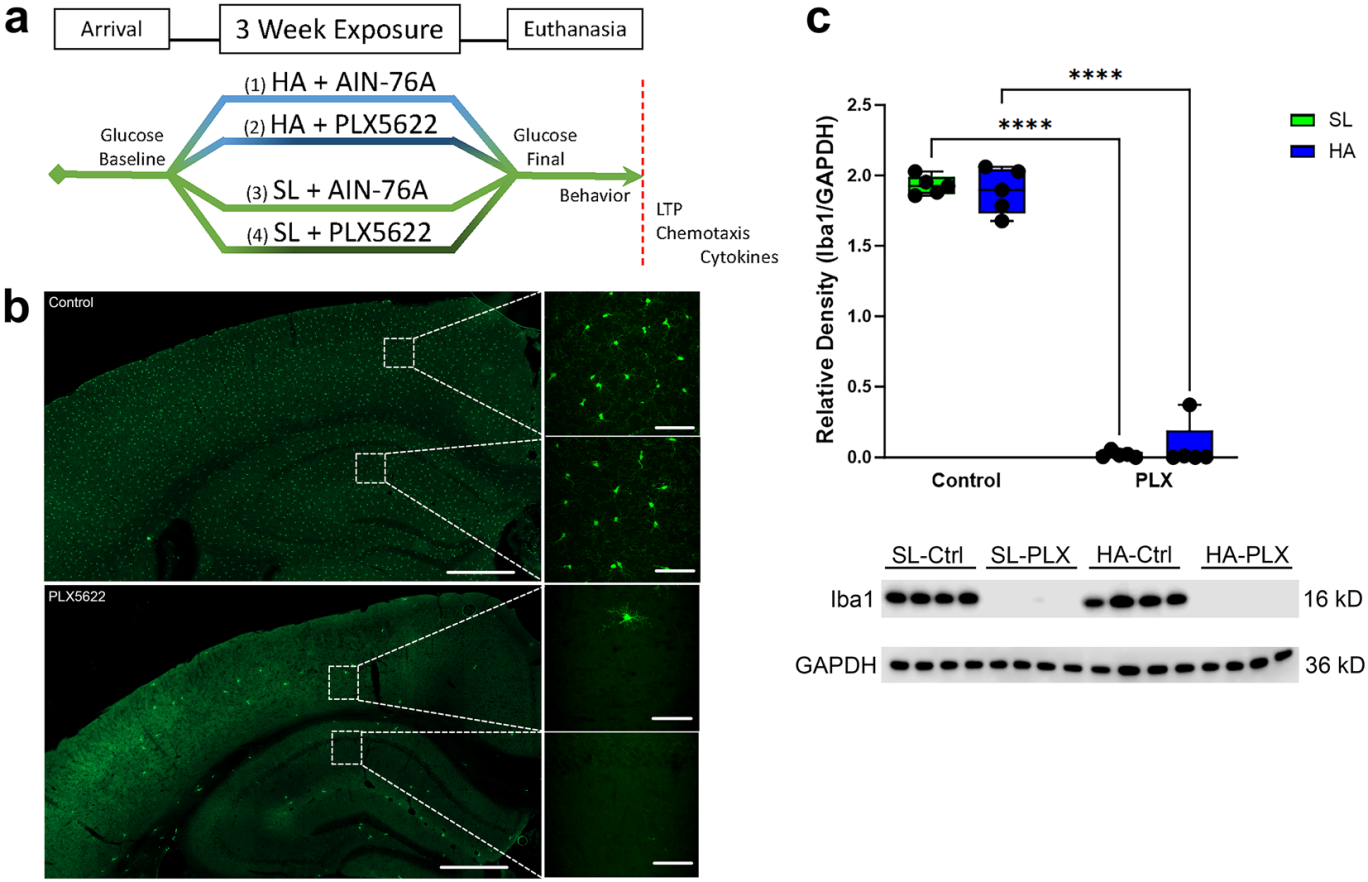
Experimental paradigm and microglia ablation. (**a**) Mice were exposed to HA or SL for 3-weeks, during which time they were fed either PLX5622 (PLX) diet (microglia-depletion) or AIN-76 control (Ctrl) diet. Microglia chemotaxis experiments were only performed on groups 1 and 3. (**b**) Representative coronal slices from CX3CR1-GFP^+/−^ mouse brains demonstrating microglia-depletion (top) compared to controls (bottom). Scale bar = 500 µm for low (10×) magnification; scale bar = 50 µm for high (40×) magnification insets. (**c**) Western blotting of hippocampal homogenate for IBA1 normalized to GAPDH confirms microglia-depletion following PLX administration for SL and HA mice (representative full blots available in Supplementary file). ****p < 0.0001, n = 5 mice per group.

It has been shown previously by our group that mice exposed to HA for a period of 3 weeks or more exhibit impairments in hippocampal-mediated memory recall along with changes in hippocampal and cortical vasculature and phagocytic microglia activity^6^. In addition, it is known that HA robustly alters neurovasculature and promotes microglia activation and that microglia physiologically interact with the neurovasculature. However, whether HA disrupts context dependent microglial function and leads to pathophysiological interactions within specific neuronal and vascular networks remains to be elucidated. Given the role of the hippocampus in cognitive function and given this region is known to lose vascular density with aging^29^ and is poorly vascularized in comparison to cortex (see review^30^), we hypothesize that microglia movement dynamics would be differentially impacted in the hippocampus and cortex of mice exposed to HA relative to sea level (SL) controls. To address this hypothesis, our study employed pharmacological depletion of microglia with the colony-stimulating factor 1 receptor (CSF1R) inhibitor PLX5622 (Fig. 1) for 3-weeks while at HA in male C57BL/6J mice to assess the contribution of microglia to HA associated cognitive deficits in fear memory recall and alterations in synaptic plasticity (LTP) within the hippocampus. As shifts in inflammation and metabolic status of mice can impact LTP, cytokines (central and peripheral) and peripheral glucose levels were measured to evaluate the impact of HA on these important facets of the microenvironment following hypoxic exposure. Finally, a cohort of heterozygous CX3CR1^+/GFP^ mice maintained at HA for 3-weeks was utilized for ex-vivo 2-photon imaging and a novel 4-dimensional (4D) quantification of microglia tip dynamic analysis to evaluate microglia function in response to microglia or vessel ablation.

## Results

### Successful depletion of microglia with PLX5622 administration

**Figure 2:**
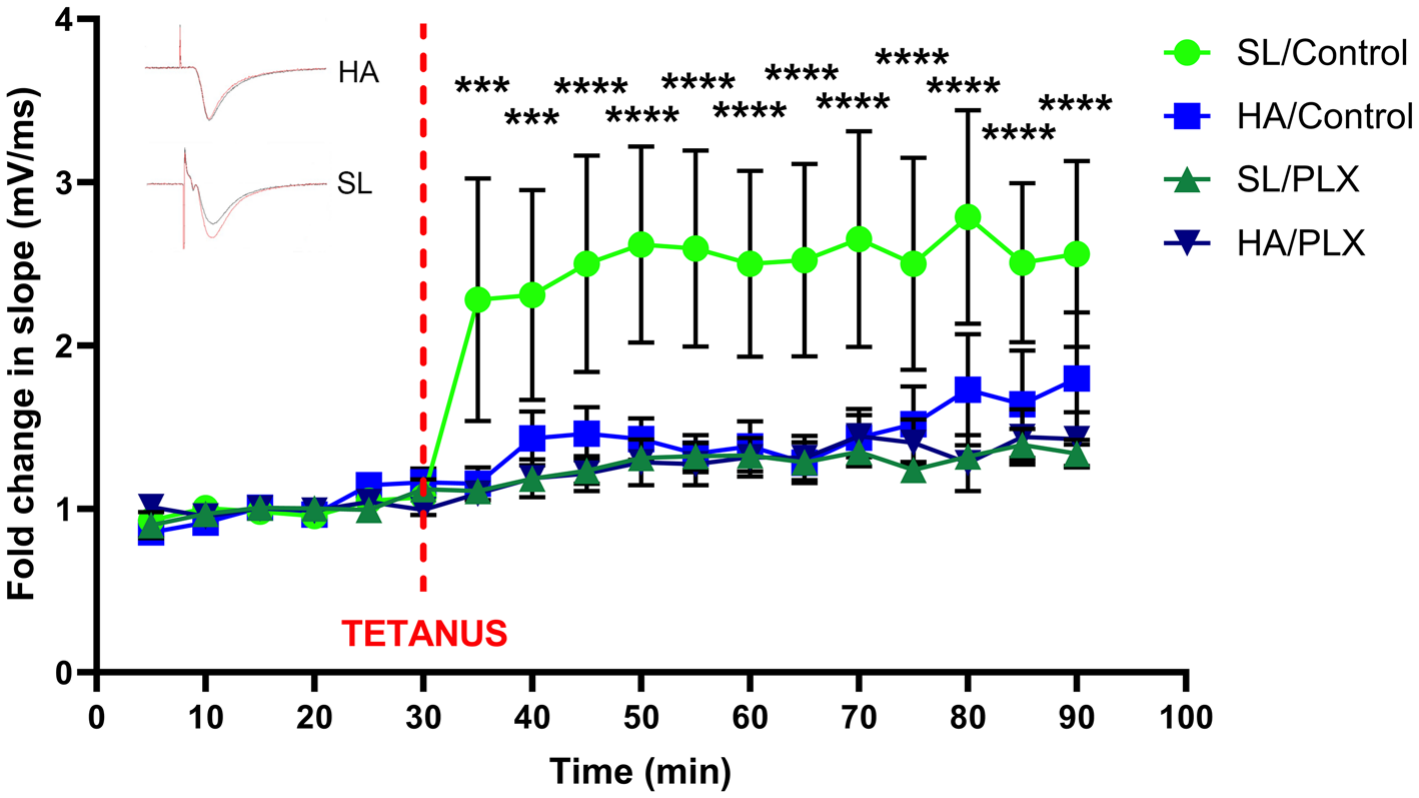
Reduction of CA1 hippocampal LTP after 3-weeks of HA exposure. There was a significant reduction in percent slope change in LTP following 3-weeks at HA compared to SL. Microglia-depletion with PLX in SL mice induced a similar reduction on percent slope change as HA. (Inset) Sample pre- (black) and post-tetanus (red) (tetanus: 100 Hz, 1 s duration; Schaffer collaterals stimulated with electrode placed in CA3 area) traces at SL and HA control diet mice. Data represents mean ± SEM, ***p < 0.001, ****p < 0.0001, n = 6–14 mice per group.. These results suggest that microglia are necessary for LTP induction, and that HA impairs synaptic plasticity.

As expected^31,32^, PLX robustly diminished the number of GFP-labeled microglia in the brains of male heterozygous CX3CR1-GFP^+/-^ mice at both SL and HA (Fig. 1a,b). Microglia-depletion was confirmed further through quantification of protein levels of Ionized calcium-binding adaptor molecule 1 (IBA1), a microglia/macrophage-specific calcium-binding protein, by western blot analysis (Fig. 1c-2-Way ANOVA with main effect of PLX5622, f _(1, 16)_ = 1190, *p* < 0.0001; representative full blots available in Supplementary file). These data are consistent with previous demonstrations of the drug’s efficacy in reducing microglia number *in vivo*^31,32^.

### Reduced hippocampal synaptic plasticity after 3-weeks of HA

Given the effect of HA alone on LTP and given the effect of microglia on LTP, we sought to elucidate whether microglia-depletion directly alters LTP at HA. We measured field excitatory post-synaptic potentials (fEPSPs) in transverse ex vivo slices from hippocampus from SL and HA mice treated with control diet or PLX5622. Consistent with previous studies showing functional hippocampal impairment^6^, ex-vivo LTP was diminished in hippocampal slices from mice after 3-weeks HA, while SL mice demonstrated robust LTP (as measured by percent slope change of fEPSPs after high-frequency tetanic stimulation compared to baseline). Microglia-depletion also significantly reduces LTP at both SL and HA (Fig. 2; Mixed-effects model (REML): Time X Altitude X PLX5622, f _(17, 473)_ = 18.15, *p* < 0.0239; with Dunnet’s multiple comparison demonstrating significant increases in fEPSPs (*p* < 0.001) at each time point following the tetanus for SL/Control mice relative to their baseline). These results suggest that microglia are necessary for the successful induction of LTP ex-vivo, and that at HA other contributing inflammatory or metabolic stressors/factors impede unimpaired/normal neuronal plasticity.

### Impaired fear memory following 3-weeks of HA

**Figure 3:**
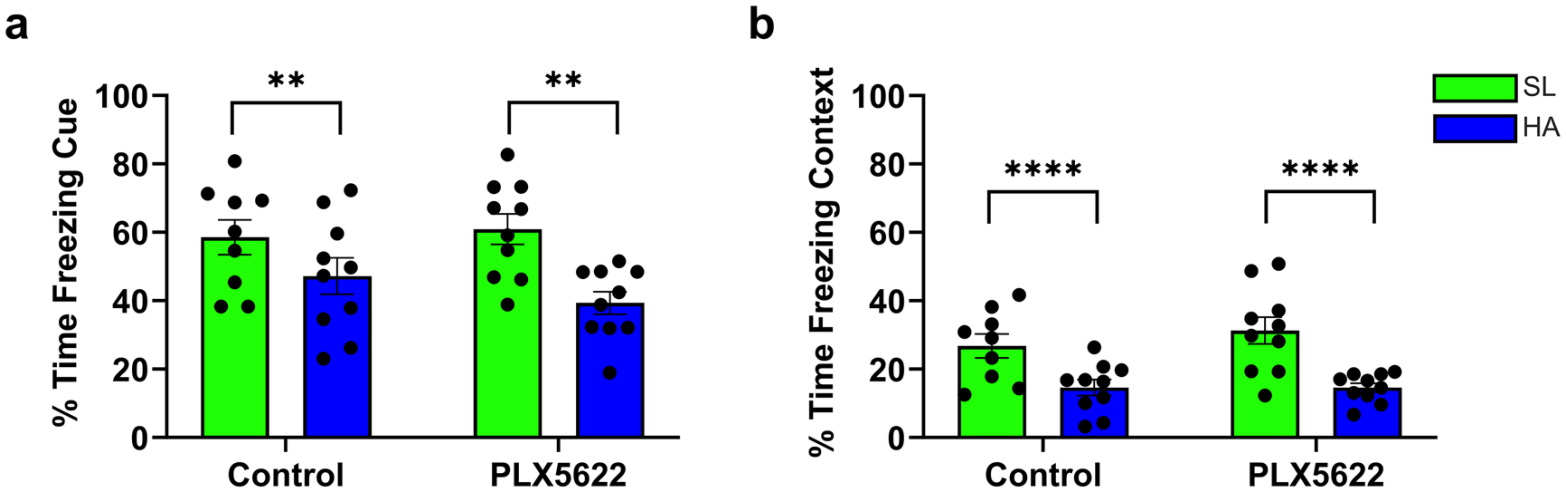
Reduction of fear memory after 3-weeks of HA exposure. (**a**) Depletion of microglia exacerbates the effect of HA on cued fear memory recall. (**b**) HA significantly reduces percent time freezing in the context condition, which is not dependent on the presence of microglia. Data represents mean ± SEM, **p < 0.01, ****p < 0.0001, n = 9–10 mice per group. These results confirm that HA impairs learning and memory, and that this is independent of microglial involvement.

To understand further functional deficits induced by HA and the role of microglia in these cognitive changes, mice were assessed using a fear conditioning paradigm following the completion of 3-weeks HA either on PLX5622 or on the AIN-76A control diet. Consistent with our previous findings^6^, HA reduced fear memory recall. A main effect of altitude was identified for both cued (f _(1,35)_ = 12.80, *p* = 0.0010) and contextual (f _(1,35)_ = 24.57, *p* < 0.0001) fear memory recall regardless of microglia-depletion (Fig. 3a,b, respectively). These results suggest that HA-triggered impairment of contextual- and fear-memory occurs with and without microglia, whereas microglia absence can be compensated by signaling pathways and/or rewiring occurring without contribution of microglia.

### Adaptation of peripheral and region-specific inflammatory cytokines and glucose after 3-weeks HA

Microglia interact with signaling factors throughout their microenvironment, which may influence response to insult and disease^33^. To understand which inflammatory factors may influence the response to HA, we assessed peripheral (plasma) and region specific (hippocampal and cortical micro dissected brain homogenates) signaling molecules, determining the levels of 19 unique cytokines/chemokines (IL-1β, IL-2, IL-4, IL-5, IL-6, IL-9, IL-10, IL-12p70, IL-15, IL-17A/F, IL-27p28/IL-30, IL-33, IP-10, KC/GRO, MCP-1, MIP-1α, MIP-2, TNFα, and IFNγ). Analysis of blood-serum cytokines or chemokines did not reveal any significant changes in the levels of pro- or anti- inflammatory molecules at 3-weeks HA. Amongst these 19 molecules, analysis of brain homogenate showed significant Altitude X PLX5622 interaction for levels of IL-15, IL-33, and IL-17A/F in the hippocampus and levels of IL-33 in the cortex. Microglia-depletion at SL increased these cytokines in the hippocampus, so too did HA alone, but no further elevation was noted in HA/PLX5622 mice (Table 1). In the cortex, HA increased IL-33 only when microglia were present, although this effect was marginally not significant after Bonferroni’s test for multiple comparisons (*p* = 0.0572). Microglia-depletion at SL and HA causes a significant increase in the level of MIP-1α chemokine in the cortex, although this increase is more robust in the SL animals (Table 1). No other cytokines/chemokines were differentially impacted by HA in the cortex. These results suggest that HA related inflammation is subtle (reflecting a continuum of intricate changes triggered by HA) and may require more targeted and precise sampling of the microenvironment to determine context-dependent nuanced effects of HA on inflammatory mediators.

**Table 1:** Hippocampal and cortical cytokine/chemokine changes after 3-weeks HA.

Region	Cytokine/Chemokine	SL	SL-PLX	HA	HA-PLX	Statistical outcome	*P* value	F (DFn, DFd)
		Average ± SEM	Average ± SEM	Average ± SEM	Average ± SEM			
**Hippocampus**	MIP-1α	3.65 ± 0.41	3.62 ± 0.44	3.47 ± 0.58	2.34 ± 0.25	ns	> 0.05	F (1, 16) > 4.49
	**IL-15**	**25.82 ± 1.31**	**34.46 ± 3.30**	**34.52 ± 2.05**	**31.51 ± 2.49**	**HA X Diet interaction**	***0.0452***	**F (1, 16) = 4.720**
	**IL-33**	**47.84 ± 4.42**	**85.22 ± 7.86**	**81.76 ± 2.89**	**75.80 ± 6.59**	**Main effect of Diet**	***0.0270***	**F (1, 16) = 5.930**
						**HA X Diet interaction**	***0.0040***	**F (1, 16) = 11.29**
	**IL-17A/F**	**0.69 ± 0.01**	**0.95 ± 0.08**	**0.87 ± 0.03**	**0.94 ± 0.05**	**Main effect of Diet**	***0.0135***	**F (1, 16) = 7.695**
**Cortex**	**MIP-1α**	**2.59 ± 0.09**	**5.02 ± 0.39**	**2.18 ± 0.09**	**3.81 ± 0.11**	**Main effect of HA**	***0.0042***	**F (1, 16) = 11.10**
						**Main effect of Diet**	** < *0.0001***	**F (1, 16) = 70.44**
	IL-15	23.82 ± 1.09	22.26 ± 1.66	26.59 ± 2.36	20.00 ± 2.32	ns	> 0.05	F (1, 16) > 4.49
	**IL-33**	**33.03 ± 2.23**	**37.82 ± 2.92**	**45.10 ± 2.62**	**36.55 ± 3.65**	**HA X Diet interaction**	***0.0352***	**F (1, 16) = 5.29**
	IL-17A/F	0.562 ± 0.04	0.636 ± 0.04	0.768 ± 0.14	0.639 ± 0.04	ns	> 0.05	F (1, 16) > 4.49

Significant values are in [bold/italic].

**Figure 4:**
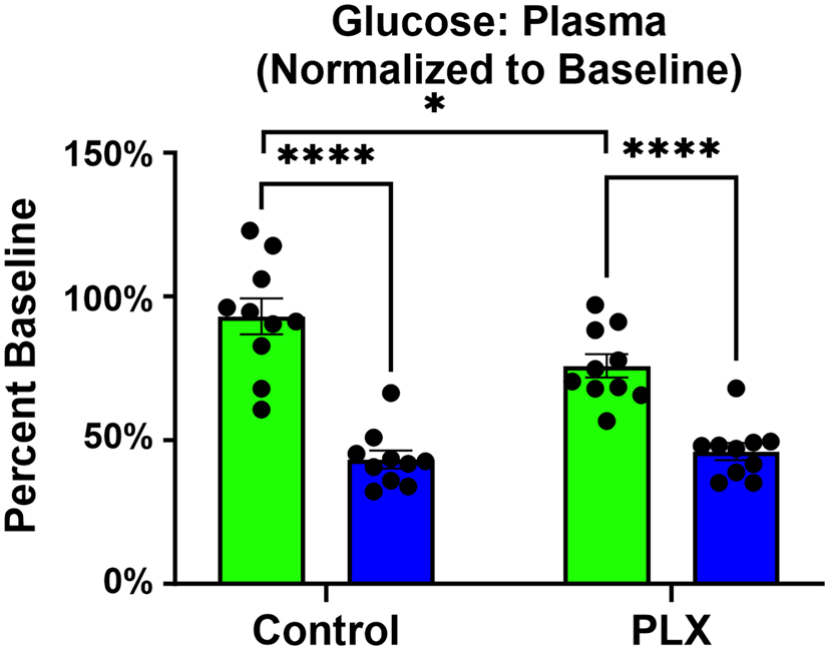
Highly reduced peripheral glucose levels show robust influence of HA. There was an ~ 50% decrease in glucose levels after 3-weeks exposure compared to baseline. At SL, there is also an impact of microglia on reduced plasma glucose levels. Data represents mean ± SEM, 2-way ANOVA with Tukey’s multiple comparisons test, *p < 0.05, ****p < 0.0001, n = 9–10 mice per group. These results show that while microglia-depletion reduces peripheral glucose levels, HA exposure has a greater impact on glucose reduction.

Glucose levels are known to affect LTP, with hypoglycemia associated with reduced LTP and hyperglycemia with increased LTP^34^. In addition, a drop in glucose and oxygen levels has been associated with “primary energy failure” in perinatal hypoxic brain injury^35^. To determine the broad impact of HA and microglia on metabolic factors, we measured peripheral glucose levels. Blood glucose levels were measured before HA to establish individual baseline values, and again after 3-weeks HA. After normalizing 3-week values to baselines, we identified a significant decrease in blood glucose of nearly 50%, regardless of microglia-depletion status (main Altitude effect: f _(1,36)_ = 85.74, *p* < 0.0001; Fig. 4). At SL the absence of microglia does decrease peripheral blood glucose levels by ~ 25% (Altitude X PLX5622 interaction: f _(1,36)_ = 5.411, *p* = 0.0258), but this does not appear to have a further cumulative impact at HA (Fig. 4). These results suggest that while microglia play a role in glucose metabolism^36^, HA causes a more dramatic impact on metabolism involving more than just microglia, but microglia may have some compounding effect on peripheral glucose level.

### Microglia movement dynamics are influenced by HA

**Figure 5:**
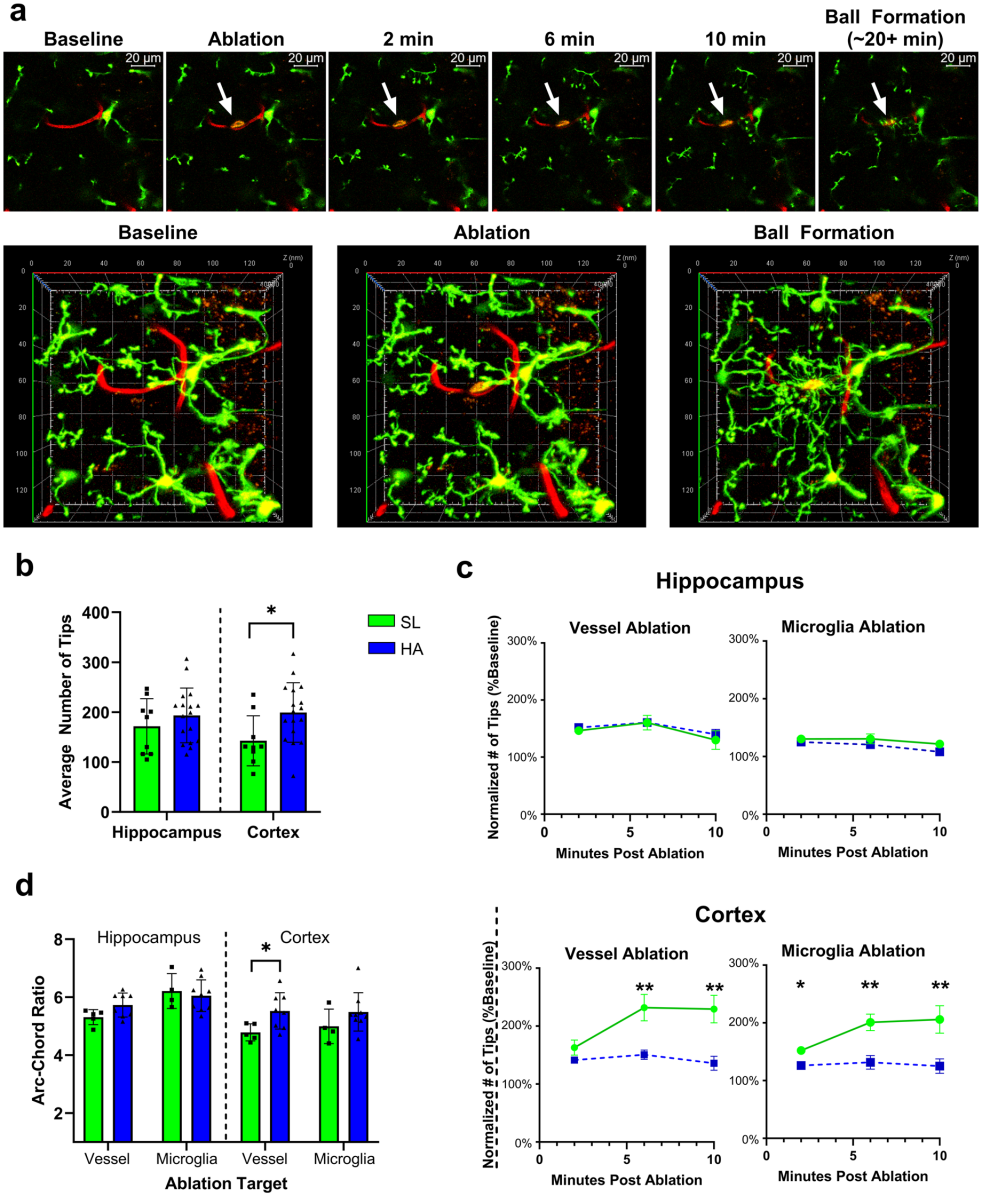
Reduced microglia tip proliferation after 3-weeks of HA. (**a**) Representative images of laser targeting of micro-vessels or microglia soma for ablation. Note the initial ball formation response from nearby microglia cells (white arrow in panel 2) indicating site of ablation, with completed ball formation in panel 6 (white arrow). (**b**) The cortex showed a greater number of microglial tips overall present during baseline surveillance activity after HA. (**c**) Microglia in the cortex following ablation exhibit reduced levels of tip proliferation after HA exposure, regardless of the nature of the ablation source. (**d**) The cortex showed increased tip path tortuosity following vessel ablation in HA exposed mice. Data represents mean ± SEM, *p < 0.05, **p < 0.01, n = 4—10 mice per group. This data suggests a muted microglial response after HA exposure.

To understand how HA may impact microglial activity, we undertook the analysis of microglia movement during homeostatic (pre-ablation) surveillance activity and activated/directed (post-ablation) chemotactic response activity in coronal brain slices of transgenic CX3CR1-GFP^+/-^ mice. This design helps us to reveal region and context-dependent changes in microglia properties, revealing region specificity of microglia activity in the hippocampus and cortex and potential novel pathophysiological vascular context in hypoxic environment. Spontaneous homeostatic surveillance activity was affected by HA in the cortex, as evidenced by an increase in the baseline number of microglia process tips identified in the cortex of mice after 3-weeks HA compared to SL (t_24_ = 2.433, *p* = 0.0228; Fig. 5b). During directed microglia chemotaxis following laser ablation experiments (example shown in Fig. 5a), we observed reduced microglia tip proliferation (assessed as number of tips normalized to baseline) specifically in the cortex after 3-weeks HA regardless of the ablation target (Vessel Ablation 6/10 min: t_11_ = 3.980/3.903, *p* = 0.0022/0.0025; Microglia Ablation 2/6/10 min: t_11_ = 2.854/3.451/3.315, *p* = 0.0157/0.0054/0.0069; Fig. 5c). At SL, greater tip proliferation in response to the ablation was observed in the cortex relative to the hippocampus, although the hippocampal microglia may be slightly more sensitive to vessel ablation than microglia ablation (Fig. 5b). We also identified an increase in tip path tortuosity in the cortex following vessel ablation in HA exposed mice (t_11_ = 2.454, *p* = 0.0320; Fig. 5d). These results support the idea that different regions contribute to the formation of unique microglial populations, and that this may impact the response to HA during chemotactic activity, possibly due to additional physiological/context factors at HA like, for instance, changes to vascularization.

**Figure 6:**
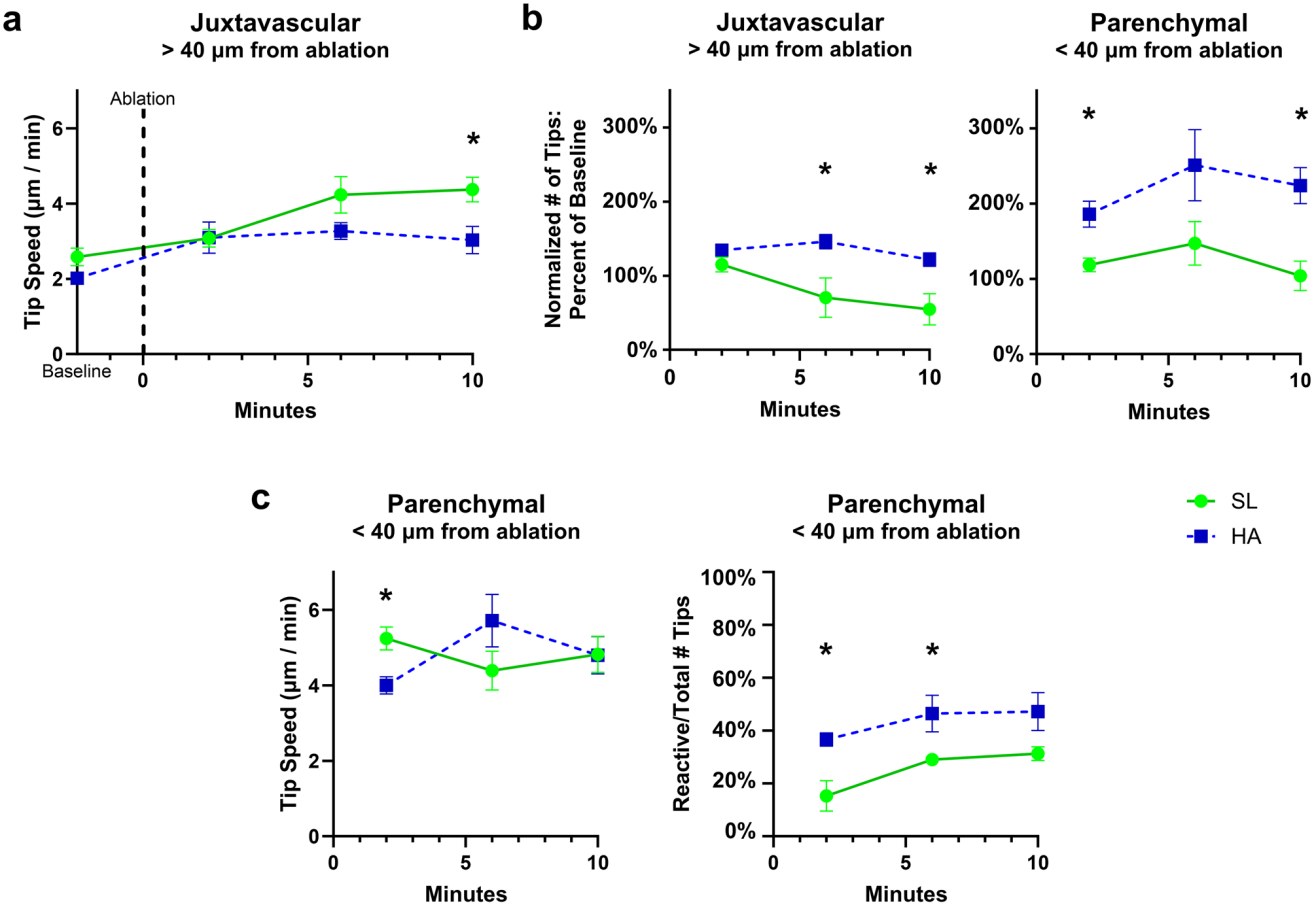
Microglial populations in the hippocampus demonstrate unique HA response to vessel ablation depending on proximity to vasculature and ablation site. (**a**) In the hippocampus among juxtavascular (< 15 µm from vessel) and far (> 40 µm from the vessel ablation site) microglia, we observe reduced tip speeds during chemotactic response after vessel ablation following HA exposure compared to SL exposed microglia which exhibit sustained increases in speed as long as 10 min post ablation. (**b**) We also find that in this subpopulation of microglia, SL microglia experience a progressive reduction in tip proliferation (or a retraction of tips) that is not replicated in mice exposed to HA, while parenchymal (> 15 µm from vessel) microglia near (< 40 µm) the vessel ablation site demonstrate increased tip proliferation after HA exposure. (**c**) These trends may be driven by the activity of reactive tips (located on the responding side of the soma and moving faster than average baseline speeds), where we see that HA exposure may delay the increase in reactive tip speed following ablation, yet increased proliferation of HA tips may be driven by a proportional increase in reactive microglia tips among parenchymal microglia close to the ablation. Data represents mean ± SEM, *p < 0.05, n = 4–10 mice per group. These data suggest microglia chemotaxis is influenced by cues in the microenvironment associated with other cells and structures.

Previous research suggests that microglia populations are unique to their regional environment^26,32,37,38^, and HA is known to induce alterations in vasculature and blood–brain barrier (BBB) permeability which may affect these micro-environmental cues^27,39,40^. To elucidate further how different microglia populations may react to HA and how neurovasculature may influence these interactions in each brain region, we categorized microglia into four distinct populations based on proximity to vasculature (1) microglia soma within 15 µm of nearest vessel = “juxtavascular”, (2) > 15 µm away from vessel = “parenchymal”, (3) proximity to the ablation site within 40 µm = “near” and 4) > 40 µm away from ablation site = “far”. We also stratified the experimental outcomes to account for the type of ablation, vessel versus microglia. This stratification was carried out in order to account for chemotactic signals, which are unique to the targeted/damaged cells. In all experiments we identified a subgroup of “reactive tips”, where the microglia soma is within 80 µm of ablation site and the tips were on the responding side of the soma with the average tip speed after ablation greater than the average baseline speed of all tips in that recording. This stratified analysis revealed some interesting chemotactic trends in the hippocampus, as well as confirming the dampened proliferation response to ablation in the cortex after HA. In the hippocampus after vessel ablation, we observed that while SL tips sustain an increased speed even 10 min into the response, HA tips specifically belonging to juxtavascular microglia far from the ablation site do not achieve the same level of response speed (t_7_ = 2.659, *p* = 0.0325; Fig. 6a). Tip proliferation is increased for this juxtavascular subpopulation of microglia in HA mice after vessel ablation (Juxtavascular Far, 6/10 min: t_6_ = 3.243/3.420, *p* = 0.0176/0.0141), as well as for parenchymal microglia near the ablation site (Parenchymal Near, 2/10 min: t_5_ = 3.728/3.914, *p* = 0.0136/0.0112; Fig. 6b). This may be influenced by the activity of “reactive” tips. In the initial period following vessel ablation (2 min), reactive tips belonging to microglia in the parenchyma near the ablation site exhibit a significantly slower response in HA exposed mice, that appears to rebound and stabilize by 6–10 min (Parenchymal Near, 2 min: t_4_ = 3.269, *p* = 0.0308; Fig. 6c), but these parenchymal microglia may compensate for this effect of HA by having a significantly greater proportion of tips categorized as “reactive” (Parenchymal Near, 2/6 min: t_5_ = 2.986/2.838, *p* = 0.0306/0.0363). These results suggest that microglial proximity to vasculature influences chemotactic responses at HA and can potentially provide structural context independently to other HA related stereotypic injury contexts.

## Discussion

**Figure 7:**
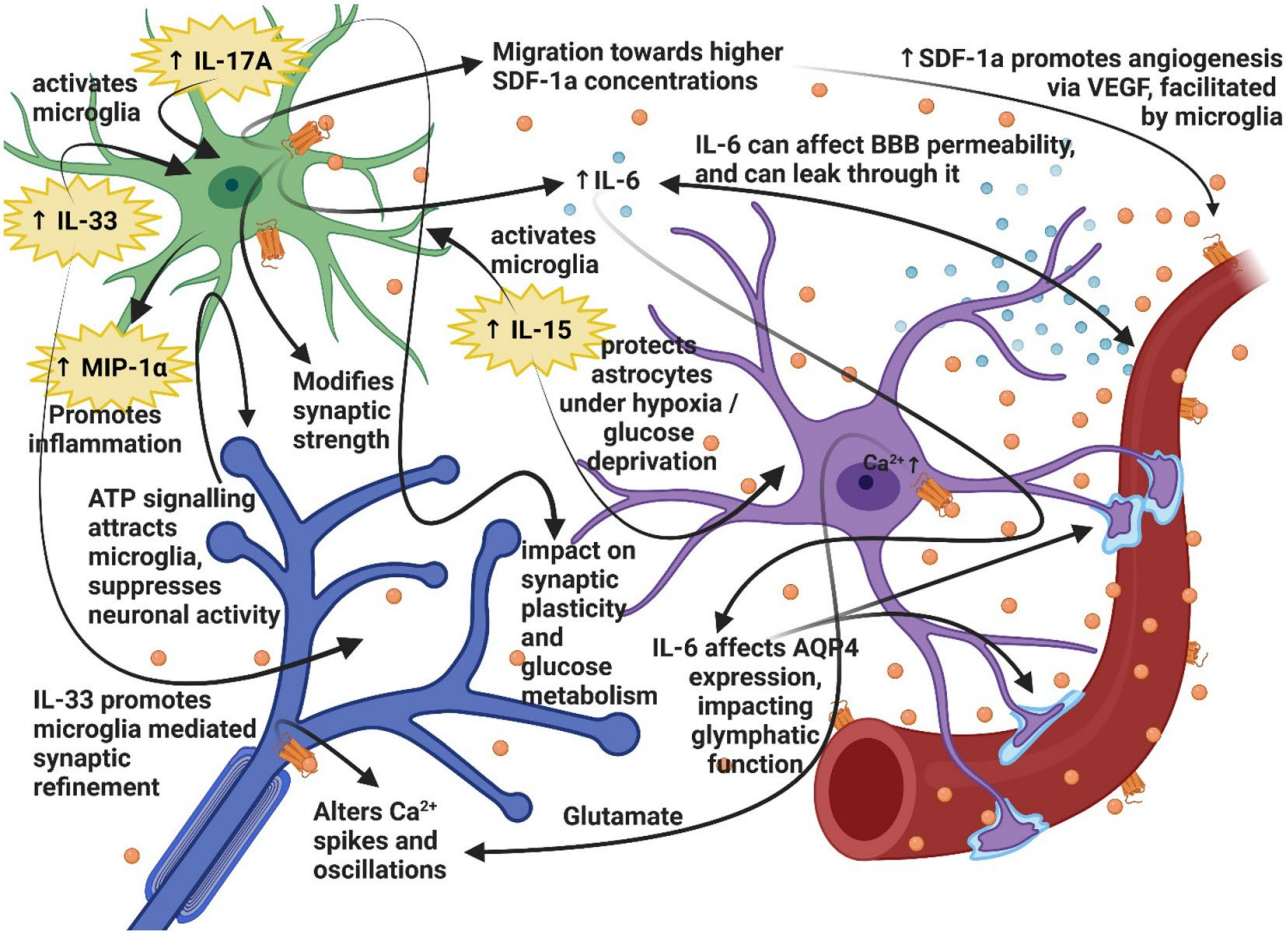
HA induces microglial activation, creating an inflammatory microenvironment impacting neuronal function. Activation of microglia (green) at HA results in the production of proinflammatory chemokines and cytokines resulting in oxidative stress and metabolic dysfunction. These changes in the extracellular signaling environment contributed to altered neuronal (blue) function and synaptic plasticity, leading to reduced LTP after HA that is consistent with observed cognitive deficits. Components of the perivascular spaces (including astrocytes, purple), and neurovascular unit are likely involved in chronic HA acclimatization. (Created with BioRender.com).

We have previously identified that chronic HA exposure promotes angiogenesis, myelination as well as microglial activation^6^. The experiments presented here explore the role of microglia following HA acclimatization. We determined that the presence of microglia is necessary for successful induction of LTP at SL. The stress of HA impairs LTP independent of microglia depletion (Fig. 2). Cognitive impairments are evident after 3-weeks HA, as demonstrated by deficits in fear memory recall, but depleting microglia did not impact the levels of recall at SL or exacerbate deficits in mice following HA (Fig. 3). Compensatory mechanisms relating to altered cytokine levels (IL-15, IL-33, IL–17A/F, and MIP-1α) and reduced glucose metabolism (Table 1 and Fig. 4) are likely to impact observed functional changes in microglial dynamics (Figs. 5, 6), including the muted chemotactic response after HA in the cortex and hippocampus following microglial and vessel ablation, respectively. It is clear that alterations to the microenvironment relating to angiogenesis and oxidative stress during HA acclimatization impact microglial and neuronal function through inflammatory crosstalk and metabolic dysregulation, which may underlie the cognitive impairment observed in those individuals that ascend to and stay at HA for prolonged periods of time (Fig. 7).

Learning, consolidation and recall of memories rely on synaptic changes relating to the induction of LTP and LTD^41^. Inability to elicit long-lasting changes in synaptic hippocampal neurocircuitry communication is caused by synaptic deficits. Here we demonstrate that microglia depletion at SL produces an attenuation of LTP that is similar in magnitude to the deficit produced by HA. For mice exposed to HA but with microglia intact, impairment of LTP could be associated with overactivation of microglia in a pro-inflammatory manner due to oxidative stress^42^. Chronic hypoxia has been shown to increase endothelial fractalkine (CX3CL1) expression and the activation of its cleaving enzyme ADAM17, which could increase the distribution of soluble CX3CL1 and disrupt the normal CX3CL1/CX3CR1 axis activity between microglia and neurons, thereby contributing to elevations in IL-1β and subsequent reduction of LTP^43–46^.

Cognitive impairment after HA exposure has been clinically reported^3,47^, and replicated in murine models of HA, measured as deficits in hippocampal mediated memory recall^6^. Our data confirm these behavioral effects, as mice exposed to HA exhibited deficits in both cued and contextual fear memory recall. While our present data show reduced LTP at SL when microglia are depleted, removal of microglia at SL or indeed at HA altered behavioral responding in this task. Given that several different brain regions including the amygdala and prefrontal cortex are implicated in the acquisition and recall of fear memory, it could simply be that this assay is not sensitive enough to detect the specific hippocampal mediated deficits. Other options going forward would be to utilize an assay that was heavily dependent on spatial cues or landmarks, such as the Morris Water Maze^48,49^, which would facilitate a more comprehensive assessment of microglia-mediated deficits in the acquisition, recall and reversal learning phases to explore landmark based navigation. Recent data from clinical study that administered minocycline in humans with normal and obese BMI scores, suggest that although microglia depletion does produce a mild to moderate enhancement of hippocampal mediated boundary navigation, the impairment in landmark-based navigation was marked in both groups, suggesting that it is changes in striatal circuitry that may correlate best with microglia depletion studies^50^.

Changes in cytokines within the extracellular environment following HA can lead to context-dependent vascular effects on microglial and neuronal activity. Increased levels of the chemotactic chemokine MIP-1α were identified in the cortex following HA and microglia-depletion. This finding implicates increased MIP-1α secretion in inducing inflammatory response and promoting homeostatic activity following HA^51,52^. Within hippocampal tissue, robust increases in the levels of IL-15, IL-17A and IL-33 at HA were detected following HA and PLX depletion in SL animals. Given the exceptionally high levels of these cytokines in the tissue, there was a ceiling to detection of PLX mediated increase in the HA animals. It has been shown previously that IL-15 plays a role in microglial activation^53^, and that astrocytes increase expression of IL-15 under oxygen glucose deprivation to protect astrocytes during hypoxic exposure^54^. IL-17A is also known to activate glial cells under inflammatory conditions, contributing to progression of neurodegeneration^55^. IL-33 signaling has been implicated in activation of microglia and cerebral endothelial cells, contributing to neuroinflammation and promoting immune cell infiltration across the BBB, as well as regulating the functional state of microglia^56,57^. IL-33 also promotes microglial mediated synaptic refinement^58,59^. It has also been demonstrated that IL-17A overexpression may contribute to a failure of synaptic plasticity and learning in a mouse model of multiple sclerosis^60^. Interestingly, IL-17A is also necessary for optimal metabolic adaptation of damaged epithelial after hypoxia towards a program of glycolytic activity^61^. Taken together, these data suggest a critical role for hippocampal IL-17A and IL33 in mediated the effects of HA exposure and microglia depletion on LTP inhibition.

In addition to shifting the brain microenvironment to a proinflammatory profile, hypoxia also affects the peripheral levels and transport of glucose across the BBB, impacting cellular brain metabolism^62–64^. This study highlights reductions in peripheral glucose levels under hypoxic conditions as well as following microglia-depletion, suggesting a role of microglia in metabolic regulation. The overwhelming reduction of glucose observed following HA in microglia-intact mice seems to have led to a floor effect, where the impact of PLX5622 diet under HA is difficult to evaluate. Going forward, alternative techniques to measure these changes will be required, to fully elicit the importance of glucose to changes in LTP and microglial dynamics. Of relevance would be 2-Deoxyglucose radionucleotide imaging under conditions of microglia depletion at SL and HA.

Microglia are known to respond to neuronal hyperexcitation, where glutamate binding to NMDA receptors on neurons facilitates calcium influx and ATP release which triggers microglial process extension^17^. In a mouse model of epilepsy, microglial motility and process velocity during basal activity was preserved even after neuronal hyperexcitation, but territory of basal surveillance was increased and directed process tip velocity towards a purinergic agonist source was elevated^23^. Our experiments analyzing microglia motility have revealed context-specific differences in microglia homeostatic and reactive activity following HA. In response to laser ablation, microglia have a greater degree of tip proliferation in the cortex at SL compared to the hippocampus, which suggests that the cortex is particularly sensitive to ablation signals. Conversely, after HA microglia show a significant suppression of tip proliferation during ball formation response activity in the cortex. This effect persists to at least 10 min post-ablation, suggesting that HA may have a priming effect in the cortex that causes microglia to maximize the number of tips participating during homeostatic activity. Thus, HA microglia do not need to proliferate because enough tips are already present to respond to the ablation. This is supported by the significantly higher number of microglia tips present during the homeostatic surveillance activity of HA exposed mice. Hypoxic stress may cause microglia to achieve a base level of activity dynamics in intrinsically and/or context-dependent manner. We further determined that for some populations of microglia, HA is associated with microglia tips traversing a more tortuous path during chemotactic activity. This may be related to a complexity of extracellular signaling gradients in the microenvironment at HA, with inflammatory cytokines and debris from BBB disruption from the increased oxidative stress at HA impacting how directly tips navigate to the site of laser damage.

Following the stratified analysis of microglia chemotaxis data, we identified a unique pattern of microglial response to vessel ablation amongst the different population categories. Specifically, HA influences tip proliferation responses in the hippocampus following vessel ablation, where juxtavascular microglia maintain their proliferative response even far away from the ablation site, and parenchymal microglia close to the ablation site exhibit heightened proliferation including an increase in percent of “reactive” tips. This may be evidence that vessel ablation creates a unique chemokine gradient that differentially impacts different populations of microglia. Those juxtavascular microglia near the ablation site may experience such a high chemokine concentration that they remain non-reactive in order to address damage on the vessel they are monitoring during homeostasis, while parenchymal microglia are left to respond strongly to the area of vessel ablation. Tips belonging to juxtavascular microglia far from the ablation site may receive enough chemotactic signal to prompt a response to monitor local vessels in the event of further vascular damage. The HA-specific response is likely related to existing signaling gradients from BBB disruption^39,40,65^, which may confuse or prime microglia chemotaxis and tip activity after HA. It has been previously reported that capillary-associated microglia regulate vascular structure and function^27^, which could further contribute to the unique activity profiles of juxtavascular microglia and microglia response to vessel ablation observed here.

The differences in microglia activity profiles seen after HA support the idea that this newly vascularized extracellular space promotes an inflammatory microenvironment which could affect glial and neuronal interactions. Integrating the findings of this manuscript with current knowledge in the field suggesting a period of leakiness associated with vascular remodeling^66–70^, we propose the following hypothesis of microglia function post HA: HA exposure is suspected to result in gross BBB dysfunction, in part due to free radical destabilization of membranes mediated by lipid peroxidation, inflammation, and activation of local HIF-1α and VEGF signaling cascades^6,71,72^. Cytokines are known to play a role in physiological processes like learning and memory within hippocampal circuitry, and various signaling factors (including IL-1β, IL-6, glutamate, are SDF-1α)^6,73^ may be critical in the interchange between neurons, microglia and astrocytes during HA acclimatization (Fig. 7).

### Limitations, future directions, and conclusions

The identification of altered microglial function in this murine model after HA exposure is compelling, but it is important to consider that the congruence with human microglia is, as of yet, unknown. Illuminating the role of microglia during HA adaptation/acclimatization continues to be a complex undertaking, due to the multifaceted and context-dependent activity profiles of microglia and the intricate impact of HA on neurovascular components. It is further complicated by the nuanced interactions of microglia with other brain cells including neurons and astrocytes, as well as the influence of gross and regional alterations to brain physiology at HA. Differences in regional microglia response to HA may be influenced by differences in neurovascular remodeling and angiogenesis during HA acclimatization, as supported by the unique microglial response relating to vessel-directed damage and proximity to vasculature. This would be consistent with increased vascularization after HA and greater vascular density in the cortex as demonstrated previously^6^. It is still unclear if changes to microglial movement dynamics are maladaptive or may be beneficial following HA exposure, and future studies are needed to resolve any simplistic notions. Moreover, while we propose that microglia show a higher degree of adaptability in well vascularized areas like the cortex, it is important to consider more factors which could play a role in addition to vessel density. One such factor to consider is the molecule adenosine, for which the receptor A1R is increased in the hippocampus after 3-weeks HA^6^, and which may provide a potential therapeutic target for pharmacological intervention. In addition to its role in promoting angiogenic phenotypes under hypoxic conditions, adenosine works to maintain metabolic balance^74^. While adenosine activation can modulate HIF-1α, its signaling also plays a role in LTP induction and regulation of microglia physiology^9,75–79^. Additional studies should also be carried out to explore the role of neuronal network elements such as neurons, myelin and astrocytes, pharmacologic or genetic inhibition or specific signaling mechanisms on microglial activity. It is also important to continue studies into the time course of acclimatization and how microglial activity may shift during the transition from acute to chronic HA adaptation phase. The data presented here on 3-week exposure is key for understanding this transitional period, providing new insights into observations from our previous investigation of the effects of 12-weeks HA. Aberrant microglial activity and the impact of inflammatory cytokine changes and metabolic disruption in the microenvironment may have a serious impact on neuronal function and cognitive outcomes after HA at different time points. Future efforts should focus on trying to reset microglia activity to restore normative synaptic plasticity by interrupting the feed forward cycle of neuroinflammation (Fig. 7), potentially through utilization of microglia-depletion or by pharmacologically targeting specific inflammatory and metabolic pathways. Utilization of PLX5622 as a pre- or post-treatment for HA might reverse maladaptive microglial activity and, if successful, could prove immediately translatable to clinical applications. We are currently in the process of conducting such experiments in our mouse model. Replication of the reported studies under normobaric hypoxic conditions would also be informative for the field, demonstrating the impact of hypoxia versus hypobaria on microglial function, which could have significant implications for conditions involving chronic hypoxia alone, such as sleep apnea.

## Methods

### Animals

Male C57Bl/6J mice were obtained from Jackson Lab (#000,664, Jackson Laboratories, Bar Harbor, ME, USA) at 7 weeks of age. For experiments requiring heterozygous CX3CR1^+/GFP^ mice, male homozygous B6.129P2(Cg)-Cx3cr1^tm1Litt^/J knock-in/knock-out mice expressing EGFP in monocytes, dendritic cells, NK cells, and brain microglia under control of the endogenous *Cx3cr1* locus (#005582, Jackson Laboratories,) were bred with female C57Bl/6J mice to produce heterozygous CX3CR1^+/GFP^ offspring. All experimental mice were group housed 5 per cage, provided food and water ad libitum and housed on a reverse 12-h light cycle (lights off at 06:00 and on at 18:00). CX3CR1^+/GFP^ mice entered the HA simulation chamber at approximately 20 weeks old (used for 2-photon experiments), while all other experimental C57Bl/6J mice entered the chamber at 8 weeks old. Mice exposed to HA were group housed in their conventional home cages, while mice exposed to SL conditions were kept in conventional cages in a separate reverse light cycle room to prevent noise interference from the chamber pump. We have previously determined that the noise attenuation within the chamber prevents exposure of HA mice to significant noise interference from the chamber pump. All procedures were approved by the Uniformed Services University of the Health Sciences (USUHS) Institutional Animal Care and Use Committee and complied with the Public Health Service (PHS) policy on Humane Care and Use of Laboratory Animals. Throughout experiments, mice were handled in compliance with the NIH Guide for the Care and Use of Laboratory Animals, ARRIVE guidelines, and applicable Federal regulations for the protection of animals in research. Experiments were performed after 3-week exposure to HA/SL conditions during which time the mice were fed either microglia-depletion diet or control diet (Fig. 1a).

### High altitude simulation

A modified Vicker’s hypobaric chamber altered by Reimers System Inc. (Lorton, VA, USA) was used for HA exposure and as previously described^6–8^. Atmospheric pressure was reduced to and maintained at approximately 7.4 psi using a vacuum pump (Welch Model 2585B or 2067B-01). The chamber achieved a simulated altitude of 5000 m (equivalent to inspired PO_2_ of 78 mmHg, 10–11% O_2_, ~ 60% SpO_2_), with ascent and descent procedures at a rate of 200 m per minute. This altitude was used based on previous identification of behavioral phenotypes in the mouse model elicited by this altitude consistent with cognitive deficits observed clinically^6,7^, specifically relating to hippocampal mediated memory impairment^5^. Chamber altitude was monitored using a digital manometer to ensure differential pressure of 7.4 psi (AZ Instrument Corp., Taichung City, Taiwan). Routine weekly cage maintenance and animal husbandry was performed at SL, and mice were monitored daily for signs of distress.

### PLX5622-mediated microglia-depletion

For experiments examining the direct role of microglia, pharmacological depletion of microglia was achieved by administration of a CSF1R inhibitor PLX5622 provided by Plexxikon, Inc. (Berkeley, CA, USA). Depletion was confirmed through imaging coronal slices of CX3CR1-GFP^±^ (Fig. 1b) and by Western blotting brain homogenate for IBA1 (Fig. 1c). PLX5622 was formulated in AIN-76A standard chow by Research diets (New Brunswick, NJ, USA) at a dose of 1200 ppm and is nutritionally identical to the control chow AIN-76A. All mice were fed either PLX5622 or control chow (AIN-76A) for 21 days while in HA or at SL.

### Western blot confirmation of microglia-depletion

Hippocampi were punched from fresh, unfixed brains and then frozen. Tissue was homogenized and stored in -80 °C until use. Upon calculation of protein concentration within the sample, protein was then denatured in Lamelli Sample Buffer using reducing agent on heat.

Samples (n = 5 for each study group; n = 20 total) were loaded on NuPage 4–12% Bis–Tris Gels (Invitrogen, NP0329BOX) and run in triplicate with 5µL/10μg of sample loaded into each well. Bis–Tris gels were electrophoresed at 200V constant for 30 min and transferred to PVDF membranes using an iBlot2 rig at 20V (1 min), 23V (4 min), and 25V (2 min). Membranes were then incubated in blocking buffer containing 5% dry milk in TBS-Tween for 1 h and placed into ionized calcium binding adapter (IBA1; 1:1000, Abcam, ab178847) primary antibody in blocking buffer overnight. On Day 2, following incubation, membranes were rinsed in TBS-T 3 times 5 min and incubated in anti-rabbit (Abcam, ab97080) HRP secondary at 1:2000 in blocking buffer. Membranes was rinsed again in TBS-T, followed by TBS, incubated in SuperSignal West Pico PLUS (Thermo Fisher, 34578), and developed on a Li-Cor C-Digit Blot Scanner using Image Studio software (v.5.2 Li-Cor Biosciences, Lincoln, NE, USA). Following developing, the membrane was rinsed in TBS, stripped, and stained against glyceraldehyde-3-phosphate dehydrogenase (GAPDH; 1:40,000 dilution, Proteintech, 60004-1-lg) as a control overnight. The protocol for Day 2 was repeated, using HRP anti-mouse secondary for GAPDH (Proteintech, SA00001-1). Protein density was measured using ImageJ (v. 1.53e, National Institutes of Health, Bethesda, MD, USA) and intensity was standardized to the GAPDH intensities for each protein.

### Hippocampal long-term potentiation

Electrophysiological assessment of LTP through fEPSPs is an established technique in the Galdzicki lab^80,81^. Briefly, mice were euthanized under heavy isoflurane anesthesia by transcardial perfusion with 25 mL of chilled 4 °C NMDG-HEPES aCSF containing (in mM): NMDG, 93; HCl, 93; KCl, 2.5; NaH_2_PO_4_·H_2_O, 1.2; NaHCO3, 30, HEPES, 20; D-glucose, 25; sodium ascorbate, 5; thiourea, 2, sodium pyruvate, 3; MgSO_4_·7H_2_O, 10; CaCl_2_·2H_2_O, 0.5^65^. Perfused animals were decapitated and the brain rapidly removed and placed in ice-cold (4 °C) NMDG-HEPES aCSF bubbled with a mixture of 95% O_2_/5% CO_2_. NMDG-aCSF reduces hypoxic damage to the brain tissue, enhancing preservation of neurons and overall brain slice health^82,83^. The hippocampi were dissected from the brain and 400-µm thick transverse slices were cut on a McIlwain tissue chopper (Brinkmann, Westbury, NY, USA) and transferred to a holding chamber for 1 h incubation in warmed 32 °C high magnesium aCSF containing (in mM): NaCl, 124; KCl, 2.5; NaH_2_PO_4_·H2O, 1.2; NaHCO_3_, 24; HEPES, 5; D-glucose, 12.5; MgSO_4_, 4; CaCl_2_·2H2O, 2, bubbled with a mixture of 95% O_2_/5% CO_2_. Slices were then transferred to a recording chamber (Kerr Scientific Instruments Tissue Recording System, Christchurch, New Zealand) and allowed to equilibrate for at least 1 h prior to recording. They were constantly superfused (~ 1–2 mL/minute) at ~ 32 °C with standard recording aCSF containing: NaCl, 124; KCl, 2.5; NaH_2_PO_4_·H2O, 1.2; NaHCO_3_, 24; HEPES, 5; D-glucose, 12.5; MgSO_4_, 1.3; CaCl_2_·2H2O, 2, bubbled with a mixture of 95% O_2_/5% CO_2_.

fEPSPs were adjusted to ~ 33% of maximal response and recorded in the CA1 region of the hippocampus with the stimulating electrode (Teflon coated platinum wire) placed in the Schaffer-collateral commissural pathway in the CA3 region. The fEPSPs were digitized using a digitizer (Model TL-1) and amplifier (Model Axon 200B) from Axon Instruments (Axon Instruments/Molecular Devices, Sunnyvale, CA, USA) and a Universal Imaging PC running WinLTP version 2.30D LTP acquisition software (Dr. William Anderson, Department of Anatomy, University of Bristol, UK)^67^. Following 30 min of baseline recordings (1 stimulus every 60 s, with constant stimulus intensity (mA) to evoke approximately one third of maximal response), post-tetanic potentiation was induced with a single train 100 Hz for 1 s high frequency stimulation and evoked responses were measured every 60 s for 1 h^80,81^. The data was processed using WinLTP^84^ and Clampfit 10.7 software (Axon Instruments, Molecular Devices, San Jose, CA, USA) (n = 6–14 slices).

### Fear conditioning behavioral analysis

Prior to fear conditioning, mice were habituated to the testing room. The experiments were conducted in sound attenuated boxes consisting of Plexiglass chambers with electrified wire grid flooring (Ugo Basile, Varese Italy). Context association was achieved through the use of black and white striped or checkered walls of the enclosure. Mouse movements were recorded using ANY-maze software (Stoelting Co., Wood Dale, IL, USA), and the parameter of interest measured for these studies was freezing behavior. During training, mice were first given a two-minute period during which time they were allowed to freely explore the chamber without a presentation of the shock. Immediately following this exploration period, mice were presented with five tone/shock pairings (0.5 mA foot shocks 2 s in duration, spaced 1 min apart), and then returned to their home cages at the conclusion of the training session. 24 h after training, mice were returned to identical chambers and context dependent memory formation was assessed by measuring the percentage of time spent freezing during an entire 5-min testing period. Cued fear memory for the tone associated with the shock was assessed in a chamber with different contextual stimuli to that of the original chamber in which conditioning was conducted. The percentage of time spent freezing in response to the cue was assessed during the tone presentation a 3-min period during the 6-min test session (n = 10 mice per group).

### Cytokine and glucose analysis

For cytokine measurements, blood was collected from the submandibular vein or terminally from the heart before perfusion. Samples were centrifuged for 15 min at 4 °C and 2000 g/rcf to separate out the plasma, and diluted twofold for analysis using Meso Scale Discovery plates (MSD, Rockville, MD, USA). For protein homogenate samples, frozen brains were sliced and the cortex, hippocampus, cerebellum were micropunched and put into T-PER (Cat#78510, ThermoFisher, Waltham, MA) with 1 × HALT protease inhibitor (Cat#87785), homogenized with a mortar and pestle, sonicated, centrifuged for 5 min at 10,000×*g* at 4 °C, and supernatant removed. Protein concentrations were determined using a BCA assay and appropriate dilutions were calculated for 100 μg total protein per 50 μL well on the MSD plates. (n = 10 mice per group, used for all assays).

Plasma and brain tissue cytokine analysis was performed using MSD V-PLEX Plus Mouse Cytokine 19-Plex Kit (Cat#K15255G, pro-inflammatory panel and cytokine panel). Samples were run as recommended by the manufacturer and read using MSD Plate Reader model 1201 (MSD, Rockville, MD). The kits measure levels of IL-1β, IL-2, IL-4, IL-5, IL-6, IL-9, IL-10, IL-12p70, IL-15, IL-17A/F, IL-27p28/IL-30, IL-33, IP-10, KC/GRO, MCP-1, MIP-1α, MIP-2, TNFα, and IFNγ.

Glucose readings were taken from the tail vein at baseline and following 3-weeks HA/SL and PLX/control exposure using FreeStyle Lite Blood Glucose Meter (Abbott, Canada). (n = 10 mice).

### Ex-vivo 2-photon imaging and novel 4D method for quantitative tip dynamic analysis

Quantitative tip dynamic analysis is a live cell imaging modality and is performed in intact brain slices perfused with aCSF. This novel method facilitates assessment of microglial dynamics in the hippocampus, which exceeds the possible depth of imaging that can be achieved through in vivo imaging of cranial windows in living mice. Briefly, CX3CR1^+/GFP^ mice were tail-vein injected with sterile filtered, undiluted DyLight 594 labeled Lycopersicon Esculentum (Tomato) Lectin from Vector Labs (Burlingame, CA, USA, Cat# DL-1177) 30 min prior to sacrifice by CO2 inhalation (to prevent an interaction of anesthetic on microglial activity). Coronal sections were cut at 400-μm thick in ice-cold (~ 4 °C) dissection solution (high-sucrose aCSF containing (in mM): KCl, 2; NaH_2_PO_4_·H_2_O, 1.25; MgSO_4_·7H_2_O, 2; MgCl·6H_2_O, 1; NaHCO_3_, 26; CaCl_2_·2H_2_O, 1; D-glucose, 10; sucrose, 206, bubbled with a mixture of 95% O_2_/5% CO_2_) on a Leica VT1200S Vibratome (Buffalo Grove, IL, USA) and incubated for 30 min at 36 °C in normal aCSF (containing (in mM): NaCl, 126; KCl, 3, NaH_2_PO_4_·H_2_O, 1.25; MgSO_4_·7H_2_O, 2; NaHCO_3_, 26; CaCl_2_·2H_2_O, 2; D-glucose, 10; sucrose, 20, bubbled with a mixture of 95% O_2_/5% CO_2_). Brain slices were kept at room temperature for up to 8 h. For imaging, the slices were mounted into a tissue slice chamber (Warner Instruments, LLC., Hamden, CT, USA) and perfused with temperature-controlled normal aCSF (~ 30 °C). A ZEISS 7MP microscope (Zeiss, Oberkochen, Baden-Württemberg, Germany) was used to perform 2-photon fluorescence microscopy approximately 80 μm below the surface of the slice. The microscope system used a pulsed infrared laser (Chameleon Vision2, Coherent, Santa Clara, CA) with a tunable wavelength range from 680 to 1080 nm and a peak power output of 3.3 W at 800 nm. Fluorescence was detected with non-descanned detectors (NDDs) equipped with a 525/50 nm bandpass for green detection (GFP) and a 605/70 nm bandpass for red detection (tomato lectin). Imaging was done using a 40 × water immersion objective (NA = 1.0) and controlled with Zeiss ZEN image acquisition software. We recorded activity in the hippocampus in *striatum radiatum* layer beneath CA1, and in the cortex in layers 3—5 of the primary motor and posterior parietal association areas (depending on slice), enabling us to perform experiments in the same slice. For microglia ablation experiments, the cell soma was targeted for maximum laser power exposure in order to destroy the cell (confirmed by loss of GFP fluorescence throughout the cell body and processes). For vessel ablation experiments, a ~ 10 μm length of target capillary was exposed to maximum laser power until the vessel ruptured. Subsequently, 20—30 frame stacks (1 μm step size) were acquired, and the 4D movement of processes analyzed. (n = 4 (SL)—10 (HA) slices).

Analysis of microglia motility and chemotactic response dynamics over time was performed using a newly-developed algorithm^85^. First, the data was registered and stabilized to uniform noise variances by nonlinear transformation. This allows for weak signals to be enhanced to improve the detection performance. The pre-processed data are then segmented to foreground and background through an iterative thresholding approach. In each iteration, the most salient regions are segmented as foreground and removed from the data and is repeated until no more significant regions can be found. Using a novel machine learning algorithm, they have developed a multi-scale microglia tip detection approach, using convex hull analysis rather than local patterns to eliminate the influence of microglia morphology changes on tip detection. This technique has the benefit of not relying on the use of deep neural networks, eliminating the need for time-consuming annotations and instead utilizing an unsupervised approach while maintaining a high degree of accuracy. The algorithm calculates geodesic distance and pixel to convex hull distance, creating a score map for tip detection. This method has a superior precision and recall compared to previously existing methods, demonstrating robust performance even in cases of substantial microglia size and morphology variation. A paper detailing the specifics of the tip detection algorithm has been published^85^.

The novelty of this algorithm is that it has the capacity to analyze our 4D data sets in a fraction of the time required for manual tip detection and without collapsing the image stacks into maximum intensity projections, providing a more accurate quantification of measures such as speed and distance. The algorithm also exhibits superior performance in precision and recall of tips compared to other peer reviewed methods in the literature^86–88^. In addition to outstanding tip detection, the algorithm maintains the relationship of tips to the parent cell for assessment of cell specific influence on tip dynamics. Furthermore, the algorithm can identify the second wavelength channel for vasculature, allowing analysis of microglia tip dynamics relative to proximity to blood vessels. This permits further analysis of unique populations of microglia, for instance, comparing the chemotaxis of juxtavascular microglia versus parenchymal microglia as performed here. For our analysis, we focused on microglial process tip speed, number of tips, and tortuosity of tip path. These metrics are indicative of microglial homeostatic activity as well as microglial response to chemokine signaling of damage due to laser ablation. Tip speed (and location in relation to the cell body) as well as number of process tips (which relates to degree of microglial proliferation) are important metrics for microglial response. In addition to these, tortuosity may be influenced by fluctuations or disruptions to the chemokine gradients, as well as physical obstructions such as increased vascular, neuronal or astrocytic density.

### Data analysis and statistics

IBA-1 expression Western blot data were analyzed using a two-way ANOVA Altitude X PLX5622 interactions followed by Bonferroni multiple comparisons test where appropriate. For the LTP data, recordings were averaged across 5 consecutive waveforms collected at 60 s intervals to compare differences in percent fEPSP slope change (mV/ms). Time X Altitude X PLX5622 interactions were analyzed using a Mixed-effects model (REML) with Dunnet’s post hoc test for multiple comparisons. Behavioral data was assessed with 2-way ANOVA for Altitude X PLX5622 interactions. Microglia chemotaxis experiments were analyzed for each timepoint using two-tailed Student’s unpaired t-test after verifying normal distribution; any instances requiring non-parametric testing utilized the Mann–Whitney test. Cytokine data and glucose readings were analyzed with 2-way ANOVA Altitude X PLX5622 interactions followed by Tukey’s multiple comparisons test where appropriate. Statistical analyses were performed in GraphPad Prism 9.3.1 (La Jolla, CA, USA) and results presented as mean ± standard error of the mean (SEM) unless otherwise noted. In all cases, significance is determined with minimum *p* < 0.05, and individual statistical tests and *p* values are noted in the results section and figure legends. Significance markers are as follows: * = *p* < 0.05, ** = *p* < 0.01, *** = *p* < 0.001, **** = *p* < 0.0001, and so forth. Data points were identified and excluded as outliers if they were more than two standard deviations from the mean.

### Supplementary Information

Supplementary Figure 1.

## Data Availability

The datasets generated and analyzed during the current study are available from the corresponding author upon reasonable request.

## Abbreviations

aCSF: Artificial cerebral spinal fluid
BBB: Blood–brain barrier
CSFR1: Colony stimulating factor receptor 1
fEPSP: Field excitatory post-synaptic potentials
HA: High altitude
HIF-1α: Hypoxia-inducible factor 1α
Hippo: Hippocampus
LTP: Long-term potentiation
PLX: PLX5622
SL: Sea level
