# Supplementary material for: The role of microglia in neuronal and cognitive function during high altitude acclimatization
Source: Sci Rep. 2024 Aug 16;14:18981. doi: 10.1038/s41598-024-69694-9 (PMC11329659; doi:10.1038/s41598-024-69694-9)
Supplement: Supplementary file 1 — Supplementary Figure 1. [file 41598_2024_69694_MOESM1_ESM.pdf]

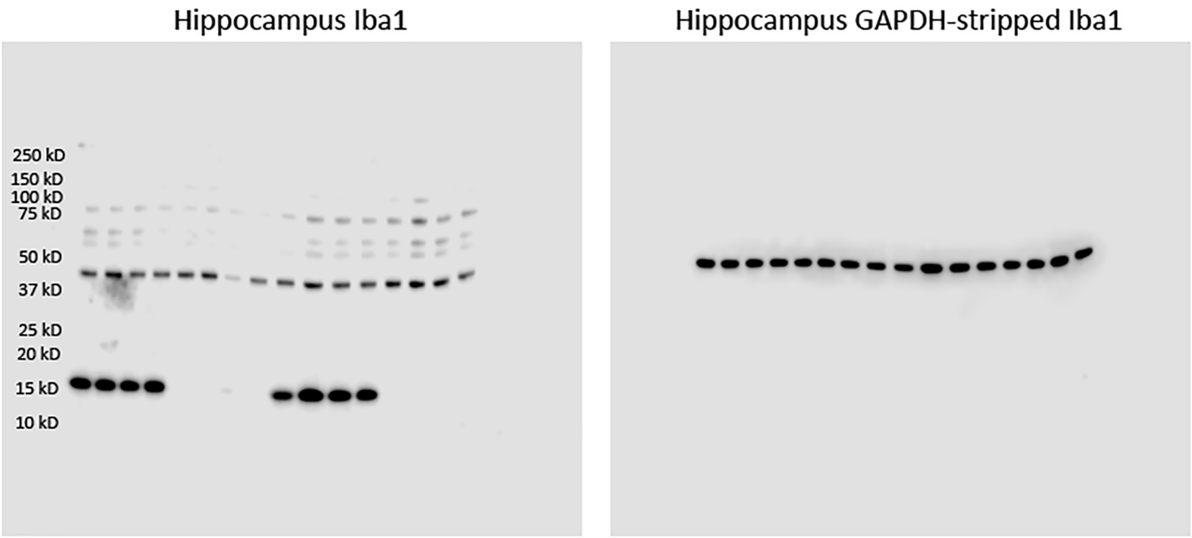

Supplemental Figure 1. Representative full Western blots of Iba1 and GAPDH presented in Fig. 1

A representative full blot image of the Iba1 staining (and subsequent GAPDH staining after stripping) for the cropped image presented in Fig. 1, demonstrating the successful depletion of microglia following PLX5622 administration.
